# Lung cancer in Hong Kong Chinese: mortality and histological types, 1973-1982.

**DOI:** 10.1038/bjc.1984.187

**Published:** 1984-09

**Authors:** I. T. Kung, K. F. So, T. H. Lam

## Abstract

The histological types of lung cancer in Hong Kong Chinese in both sexes are presented. When the 1981 WHO Classification is used instead of the 1967 WHO Classification, more than half of the large cell carcinoma is retyped into the adenocarcinoma group. The proportion of large cell carcinoma thus decreases from 14.7% to 6.9% in the male and from 10.0% to 4.4% in the female. Compared to the 1948-1962 report from the same Department, there is a shift of the peak age distribution from the 6th decade to the 7th decade. Compared to the 1960-1972 report from the same Department, there is a significant increase in the relative frequency of adenocarcinoma in both sexes, from 15.6% to 25.8% in the male and 34.3% to 49.6% in the female. Adenocarcinoma continues to be the most common histological type in females and it is becoming the commonest type in males. It is also found in the present series that in the male, the proportion of adenocarcinoma decreases with increasing age, from 60% in the third decade to 26.6% in the eighth decade. In spite of the high female lung cancer mortality rate and therefore low male:female ratio of about 2:1, there is a tendency for this ratio to increase over the years. It is speculated that factors other than active cigarette smoking may be responsible for the peculiar and changing histological pattern and the female predominance in lung cancer in Hong Kong Chinese.


					
Br. J. Cancer (1984), 50, 381-388

Lung cancer in Hong Kong Chinese: Mortality and
histological types, 1973-1982

I.T.M. Kung', K.F. Sol & T.H. Lam2

'Department of Pathology, University of Hong Kong, Queen Mary Hospital; and 2Department of Community

Medicine, University of Hong Kong; Hong Kong.

Summary The histological types of lung cancer in Hong Kong Chinese in both sexes are presented. When
the 1981 WHO Classification is used instead of the 1967 WHO Classification, more than half of the large cell
carcinoma is retyped into the adenocarcinoma group. The proportion of large cell carcinoma thus decreases
from 14.7% to 6.9% in the male and from 10.0% to 4.4% in the female. Compared to the 1948-1962 report
from the same Department, there is a shift of the peak age distribution from the 6th decade to the 7th
decade. Compared to the 1960-1972 report from the same Department, there is a significant increase in the
relative frequency of adenocarcinoma in both sexes, from 15.6% to 25.8% in the male and 34.3% to 49.6%
in the female. Adenocarcinoma continues to be the most common histological type in females and it is
becoming the commonest type in males. It is also found in the present series that in the male, the proportion
of adenocarcinoma decreases with increasing age, from 60% in the third decade to 26.6% in the eighth
decade. In spite of the high female lung cancer mortality rate and therefore low male:female ratio of about
2: 1, there is a tendency for this ratio to increase over the years. It is speculated that factors other than active
cigarette smoking may be responsible for the peculiar and changing histological pattern and the female
predominance in lung cancer in Hong Kong Chinese.

Since the late 1960s, when lung cancer replaced
liver cancer as the most important cause of cancer
death in Hong Kong, there has been a steady
increase in mortality from this malignancy in both
sexes throughout the last 15 years. The mortality
rate on 1981 of 53.3 in the male and 22.9 in the
female is more than double that of 21.7 and 11.4 in
1960 respectively (age-adjusted to world standard
population, Waterhouse et al., 1976).

Lung cancer in Hong Kong Chinese is peculiar in
that there is a high incidence in females, a fact
shared by Chinese populations in other parts of the
world, including China, Singapore, California and
Hawaii (China, 1979; Law et al., 1976; Waterhouse
et al., 1976). This results in a very low male:female
ratio of about 2:1. In addition, the proportion of
adenocarcinoma among females is high.

Material from the same department has been
reported in two separate articles, covering the
periods 1948-1962 and 1960-1972 respectively (Lee
& T'so, 1963; Chan & MacLennan, 1977). This
present study reviews the histological types of lung
cancer in the period 1973-1982 and draws attention
to the changing histopathological pattern and age
and sex incidence over the last two decades in
Hong Kong. A comparison is also made of the
percentage distributions of the various histological
types using the two WHO Classifications, (WHO,
1967; WHO, 1981).

Correspondence: I.T.M. Kung.

Received 1 February 1984; accepted 30 May 1984.

The present series is the largest series of lung
cancer cases in Hong Kong ever reported. The
observation of the difference in distribution in cell
types when using the 1967 and 1981 WHO
classification is also reported for the first time in
Hong Kong.

Materials and methods

All bronchial and lung cancer cases in the 10-year
period 1973-1982 from the University Department
of Pathology, Queen Mary Hospital were reviewed.
Rare lung tumours such as carcinoids, adenoid
cystic  carcinoma,   mucoepidermoid   tumour,
sarcomas and metastatic carcinoma were all
excluded from this study. The tissues came from
surgery and autopsy. Only Chinese patients were
included.

Surgical material included bronchial biopsies,
transbronchial lung biopsies, needle biopsies, and
resection specimens. Biopsies of lymph nodes alone
were not included. The majority of the specimens
came from the thoracic units of medical and
surgical departments of the Queen Mary Hospital
which is the major general hospital of Hong Kong
Island, and from the Grantham Hospital which is a
cardiothoracic centre. A minority of specimens
came from private clinics.

Rigid bronchoscopy was the chief method for
obtaining biopsies before 1978. Since then this was
substantially replaced by fibreoptic bronchoscopy

?) The Macmillan Press Ltd., 1984

382      I.T.M. KUNG et al.

which allowed both bronchial and transbronchial
lung biopsies. Patients with both biopsy and
resection were included in the resection series.

Autopsy material covered all necropsies on lung
cancer cases during the period in the Queen Mary
Hospital, except those performed for medical-legal
purposes. Cases that appeared in both the surgical
and the autopsy series were included in the analysis
of the surgical (resection) series, so that all the
cases were analysed chronologically according to
the year of diagnosis.

All the original histological sections were
reviewed, with no knowledge of sex, age of the
patients and the reported diagnoses. Histological
typing was based on WHO International
Histological Typing of Lung Tumours. Both the
1967   and   1981  classifications  were  used
simultaneously. The criteria were strictly followed
and adopted by both of the pathologists (ITMK &
KFS). Whenever there was a suggestion that the
tumour could be an adenocarcinoma, Alcian Blue
and Periodic Acid Schiff stains were performed for
identification of both acid and neutral mucin.

The tumours were grouped into the four major
types, i.e. squamous cell (epidermoid) carcinoma,
small cell (anaplastic) carcinoma, adenocarcinoma,
and large cell carcinoma. No subtyping was
attempted. and bronchiolo-alveolar carcinoma was
included in the adenocarcinoma group. There were
four   cases  of  combined    squamous   and
adenocarcinoma, two in either sexes. They were
grouped under the heading "Others". Biopsies,
mainly transbronchial ones, that were adequate for
diagnosis of malignancy bur insufficient for typing
were also put into the "Others" group.

Cases without histological examination of the
primary tumour of the lungs, or were diagnosed by
cytology alone were excluded from the series.

adenocarcinoma in males is increased from 25.8 to
33.6 and in females from 49.6 to 55.1. This increase
is due to reclassification of 53.3% and 55.9% of the
cases in the male and female respectively from
being classified as Type IV according to the 1967
Classification to Type 3 according to the 1981
Classification. The main difference between the two
classifications in this respect is the inclusion of solid
large cell carcinoma with mucin secretion into the
adenocarcinoma group in the 1981 Classification.
The figures for epidermoid (squamous) carcinoma
and small cell (anaplastic) carcinoma remain the
same in both classifications.

The sex and age distribution of all 1055 cases of
lung cancer irrespective of type is shown in Figure
1. The largest number of cases occurs in the 7th
decade, followed by the 6th decade in the male and
the 8th in the female. The overall male: female ratio
is 2.1:1. The male: female ratio is higher than 2.1:1
in and before the 7th decade and is highest in the
6th (3.5:1). After the 7th decade, the ratio
diminishes and is less than 1 in the 9th decade and
after.

The mean age of male patients is 60.5 years and
that of female patients 64.4. Using the WHO 1981
Classification, the mean ages of males in years in
the four main types are: 61.3, in squamous
carcinoma; 62.8, in small cell carcinoma; 58.4, in
adenocarcinoma; 60.6, in large cell carcinoma.
Those in females are 64.8; 67.8; 63.6; and 63.0
respectively.

The percentage distribution of histological type
by age group and sex is shown in Table II. In
males, there is a trend for the proportion of
adenocarcinoma to decrease with age. In females
however, the trend is not obvious.

Discussion

Results

There were 636 biopsies, 175 resection specimens
and 263 autopsies. Nineteen of the autopsy cases
appeared in both the surgical and the autopsy
series, and were included in the analysis of the
resection series. The rest of the autopsy cases had
no biopsy or ante-mortem medical or surgical
treatment. There is thus a total of 1055 cases: 714
males and 341 females. The three sources of tissue
were combined for analysis of the percentage
distribution of histological types, as this should
reflect better the overall picture in life. The
histological types by year and sex are presented in
Table I.

When the 1981 WHO Classification is used
instead of the 1967 Classification, the percentage of

As in many Occidental countries, there has been a
steady increase in the incidence and mortality rate
of lung cancer in Hong Kong over the last twenty
years. Despite the influx of immigrants from
Mainland China, (which are usually young males),
the total number of lung cancer cases and of lung
cancer deaths as well as mortality rates (crude or
age adjusted) have been increasing. The increasing
rates are due to the greater increase in the total
number of lung cancer deaths than the increase in
population. The increasing trend is also reflected in
the present series. There was a 60% increase in the
average number of cases per annum in both sexes
when compared with the series by Chan &
MacLennan (1977), covering the period 1960-1972.

Compared to Occidental countries, the incidence
in males is not striking. Hong Kong ranks only

LUNG CANCER IN HONG KONG 1973-1982  383

0% r 0% N - W) I'D I-D "

-~ (N- "     ,~tR 0~m

0  r  00: c- (NI (NI ~6

U~ 0   wi 00 '/6 -l eC(i

-(N

o  z Ni C-0  06 -000 N. 0%
Ci) tO (NI  v N  tn N 0

(N       r    r00 N   10~0 ~0%

O -c- =RtOetiN,4r

tr l~0% 'f-; N- ~16 (4 cr  00

N0000N0  0     N (IC 0%   0%

r- n ~0 0%0%0

0~ ~6~6 r r-~~16  ~ r-- tri(NI
1-   -   -   -   -11 11 11 1~

0' 0  vn it  t N -e4  o 0

c  i?c ii tri c  o6   -

C' -4 -4  - 4  -4 -q "

t     0 c 'it m " 0 N
-c - i  cj -6 wi ,f i

oC       k N  n  oo 0 0  N
" M         enO tr en N ?

00 3N- 0 br O'~ - N0 0000

?    ~   - N 0 0- 00  c i
NN    , N N N NN 0 00 C.,

0% 0% -% 0% 0   %   0%

?

n- N

en N

o N
6 .6

4 -.

-

I-0

0 0)
~0

0
~ci

o ,c
OCO

~0 )

- cd

COd

0

-o

0

U

Ut

0.

00

N

0 o

%0%

CZ

.2

U

to

0

C0
;0
0:
Z )

~0

CO
_
U)

C'

~.o N"

-q N
Nt ~

-

r-
N
CO
cr.
0
U
I a

u
CO
r.

CO
0

*N-

IN
20%

0%4

I00

0%
11

N oo

00 ~o
cri tl

ci r-

en Fo

0 E0

N
11

384      I.T.M. KUNG et al.

258

201

128

97

(A
C,

C.;
C

C

2

30

7

20- 29  30- 39   40- 49

50 -59   60 - 69

70 -

66

Age (y)

Figure 1 Sex and age distribution of 1055 cases of lung cancer irrespective of type. (3) male; (l) female.

twenty-first in male lung cancer mortality in the
world in 1975 (Segi et al., 1981). The more notable
feature is in the female. The mortality in Hong
Kong Chinese female is highest in the world (Segi
et al., 1981), possibly second only to the Maoris in
New Zealand (Waterhouse et al., 1976). This results
in the strikingly low male to female ratio which is
about 2: 1. In Occidental countries, the male: female
ratio has been high. In 1960, the ratio in Scotland
and USA were 7.6 and 6.6. respectively (Segi et al.,
1981). Since then there has been a decrease in this
ratio as lung cancer in the female gain more
importance. In Hong Kong, although the
male:female ratio has been outstandingly low, there
is a tendency to increase instead of the contrary.
The ratio calculated from mortality rates, was 1:1
in 1960 and has increased through 1.5 in 1972 to
1.8 in 1981 (Hong Kong, 1960-1981). This gradual
increase in male:female ratio is reminiscent of the
trend before 1960 in European countries and the
United States. This is perhaps because cigarette

smoking has not really become popular until after
the Second World War for economic reasons.

The male:female ratio for the period 1973-1977
in the present series is 1.96:1 and that for 1978-
1982 is 2.17: 1. This series thus also reflects the
trend in the whole population. It is also interesting
to note that in Japan there is also a tendency for
the male:female ratio to increase in recent years
(Segi et al., 1981).

In the present paper, we hope to reflect the
changes in histological types in Hong Kong by
studying material in our Department collected at
different times. It may be argued that results from
one department may not be representative of the
whole colony. However, while proportional
distribution in lung cancer cell types and its secular
trend should ideally be studied on series derived
from population-based cancer registry, it is not
practicable in Hong Kong because more than half
of the lung cancer cases notified (voluntarily) to the
registry were not confirmed histologically. Data on

(n

I

LUNG CANCER IN HONG KONG 1973-1982  385

N m o~ N
N n t  e

a-, 00 01
mI~ N - o 6 0  0-

0- t  r- 0)W   q I C 0

000 ? N 0 o
o   t on  o

c4   C-  - C

0 ct 00  tNo

m ei 000 o ei

e1 ~0Ot) -1 C

1 0 0 ') N

o o  m   ii o0o

n - 0 e e   o

O 00 N ?) 0) -

C J 1 -

0O N -00 00- N

6  x0 0  m- (:  -

---m I

c.  .6 .  .

O   C14 O 1 00

0I en      ;  I X0

00

6

00

cell types for the whole of Hong Kong and cell type
specific rates are not available nor will they be very
reliable because of the variation in standards and
procedures of clinical and/or pathological diagnosis.
However, if the proportional distribution of cell
types can be accurately assessed, cell type specific
rates can be calculated if the total number of cases
and population data are available for the
community as a whole. Opportunity for comparison
is also available because there were data on 2
previous series of lung cancer cases during 2
different periods (1948-1962) (1960-1972) from the
same pathology department. In the present paper,
more detailed comparison is made between the
1960-1972 series and the present series (1973-1982).
Comparability is maximised because we have the
same source of cases and we use the same
classification system. Moreover, the same exclusion
criteria were adopted. Cases diagnosed with
cytology and cases without histological examination
of the primary tumour were excluded in both series.

The exclusion of the latter category is inevitable
since malignancy cannot be confirmed and data on
cell type is lacking. A bias may have however be
introduced if some particular groups are not subject
to any form of histological examination because of
extraneous factors such as poverty and uneven
distribution of medical care. The present series was
derived from a pathology department which serves
mainly the two most important hospitals for lung
cancer on Hong Kong Island: one regional (and the
only teaching hospital until 2 months ago) and one
specialist chest hospital. These two hospitals are
government (assisted) hospitals and the cost of
hospitalisation is minimal (about ?1 per day). All
investigations, including pathology and treatment
are free of charge. For lung cancer patients from
Hong Kong Island, most of them are admitted,
diagnosed and treated there, poor and rich alike.
There are only a few rich patients being treated in
private hospitals which only constitute a small
proportion of all hospital beds in Hong Kong.
Compared to the rest of Hong Kong, the
proportion of lung cancer patients not being
confirmed pathologically are much less on Hong
Kong Island.

Apart from using the same criteria of excluding
cytology cases as the previous series, another reason
was because there is not yet an internationally
popular classification for lung cancer cytology
comparable to the one for histology by WHO. As a
result a large proportion of cases is unclassified.

This is shown in the results of Lam and
associates' series of 480 patients treated in the same
teaching hospital as the present pathology
department, which included 112 cytology cases;
35.7% (40 cases) of the cytology cases, were

N

00

C)
0
B

~-0

C)

. _

CO

(U

CO

0- )

o0S

x

911

PI",

0
C)

00

(U

00
0

o
00

0

00

CA

CO

0
0)
,0

0

0

C-c

0

:F

o

1-4

0E

0

0)

00

CO

0
0)

386      I.T.M. KUNG et al.

unclassified. The overall unclassified proportion
was also high, 13.1%. If the cytology cases were
excluded, the proportion would be reduced to 6.3%
which would be quite comparable with the present
series.

In an earlier series by Chan et al. (1979), both
cytology cases and cases with only radiological and
clinical diagnosis were included and the unclassified
proportions were even higher; 15.4% in males and
30.7% in females. This series of patients were
drawn from all over Hong Kong.

Table III shows the percentage distribution in cell
types in 5 series of lung cancer in Hong Kong.
Both the 1976-1977 and the 1976-1980 series when
compared with the 1960-1972 series shows that
there  is  an   increase  in   proportions  of
adenocarcinoma and supports the findings of the
present series.

Finally, Lam et al. (1983) showed that survival in
squamous cell carcinoma and adenocarcinoma cases
was quite similar. This therefore excludes the bias
due to the difference in prognosis of two types of
lung cancer which may affect the proportional
distribution.

With these background in mind, we compare
briefly our results with those of Lee & T'so (1963)
and in more detail with those of Chan &
MacLennan (1977).

There are several differences between the present
series and the series of Lee & T'so (1963) from
1948-1962 when lung cancer was not so common.
Male predominance was not noticed until the fifth
decade in their report, and the peak incidence was
in the 6th decade. Adenocarcinoma was the most
common histological type in both sexes, 34.8% in
the male and 46.7% in the female. Although their

materials were obtained from the same department
as the present series, because their case number was
relatively  small  (n = 228)  and  a  different
classification was used, comparison and inference
on time trend is not very reliable.

More reliable comparison, however, can be made
with Chan and MacLennan's results (1977), because
in addition to using materials from the same
department with the same catchment area, they
used the same classification, i.e. WHO 1967
Classification. Their case number was also larger
(n=853). We also use the same exclusion criteria as
they did. There is an obvious increase in proportion
of adenocarcinoma in the male from 15.6% to
25.8% (P<0.02) and in the female from 34.3% to
49.6% (P<0.01). The increase in adenocarcinoma
is accompanied by a drop in percentage in
squamous carcinoma, but not small cell carcinoma
in the male. The opposite is true with the female,
the increase in adenocarcinoma is associated with a
decrease in small cell carcinoma but not squamous
carcinoma. The proportions of large cell carcinoma
remain relatively constant in both series (Table I).
It is unlikely that there should be a significant
inter-observer variation as the same classification is
used, the sections were reviewed blind, and the
changes are in squamous, small cell and
adenocarcinoma which are unlikely to be confused
with one another, in contrast to large cell
carcinoma.

In this paper, we also compare the results
obtained when different WHO classification, 1967,
1981, were used. It is useful in quantifying the
change in proportional distribution of cell types
when a new system is used. In Hong Kong, the new
system is not commonly used yet. However, such

Table Ill Percentage distribution of histological types in 5 series of lung cancer in Hong Kong.

Males                                  Females

Series in                                            Total                                   Total

chronological order        I     II   III   IV    Others number   I     II   III    IV   Others number
1. 1948-1962a            22.5   17.4  34.8  20.3   5.1    138    14.4   7.8  46.7  26.7    4.4     90

(Lee & T'so)

2. 1960-1972a             43.6  21.5  15.6  15.8    3.5    576   22.7  23.8  34.3  16.2    2.9    277

(Chan & MacLennan)

3. 1976-1977b             43.3  12.5  21.6   5.3   15.4   208    23.8   9.5  33.9   2.1   30.7    189

(Chan et al.)

4. 1976-1980c             43.5  12.7  22.2   8.3   13.3    315   30.3   9.7  43.0   4.2   12.7    165

(Lam et al.)

5. 1973-1982a             33.3  21.3  25.8  14.7    4.9   714    22.6  12.6  49.6  10.0    5.3    341

(Kung et al.)

aFrom the same pathology department serving one teaching and one chest hospital.

bFrom all over Hong Kong; including cytology and X Ray/clinical cases. The category of "mixed type" is not listed
here.

cFrom the same teaching hospital as above; including cytology cases.

Note: In the 2nd to the 5th series, 1967 WHO classification was used.

LUNG CANCER IN HONG KONG 1973-1982  387

quantification is useful because adjustment can be
made when comparing 2 series classified on
different systems especially when the new system
has replaced the old one in the future.

The tendency for adenocarcinoma of the lung to
increase has been noticed in three other reports
(Vincent et al., 1977; Cox & Yesner, 1979; Valaitis
et al., 1981). The increase in these studies is mainly
due to increase in men. In the present report, the
increase is seen in both sexes, and in fact more
significantly in women.

This changing histopathological pattern of lung
cancer raises again the question of the association
of tobacco smoking with histological types.
Although the concept that adenocarcinoma is not
related to smoking has been popular since Doll et
al. (1957) and Kreyberg (1962) published their
reports, this view has been disputed by other
investigators (Kennedy,  1973; Belcher,  1975).
However, studies in Hong Kong and in Singapore
on Chinese patients, show again that smoking is
related to squamous carcinoma and small cell
carcinoma but not adenocarcinoma, particularly in
women (Chan et al., 1979; MacLennan et al., 1977).
In fact, 61% of the females with adenocarcinoma
did not smoke (Lam et al., 1983).

The kerosene stove at one time was thought to be
the cause of lung cancer in Chinese women (Leung,
1977). This association however has been shown to
be weak (Chan et al., 1979).

Although Schoental & Gibbard (1967) found
carcinogens in Chinese incense smoke, Buddhist
monks and nuns who presumably should be most
heavily exposed have not been known to have a
high incidence of lung cancer.

Recently, several reports suggest that passive
smoking may be a cause of lung cancer in women
(Hirayama, 1981 & 1983; Trichopoulos et al.,
1981). This is being investigated in Hong Kong (by
THL, ITMK and others).

Up to now, the cause of adenocarcinoma of the
lung in non-smoking women in Hong Kong
remains unknown. Investigations into environ-
mental factors so far has been inconclusive.
Although attention is mainly focussed on lung
cancer in female in Hong Kong because of the high
proportion of non-smokers among the patients with
the predominating adenocarcinoma, it is important
to  realize  that  there  is  an  increase  of
adenocarcinoma in males also over the years.
Moreover, 22% of male adenocarcinoma cases were
non-smokers compared to 4% and 3% in
squamous and small cell carcinoma respectively
(Lam et al., 1983). The reason for this increase in
adenocarcinoma in both males and females is at
present uncertain. It is possible that the same
aetiological agents operate in both men and
women. Environmental and genetic factors may act
synergistically. This latter argument is perhaps
supported by the observation that adenocarcinoma
patients in both sexes are generally younger than
those  with   other  histological  types.  Aryl
hydrocarbon hydroxylase level in various tissues in
lung cancer patients is being investigated (by ITMK
and others).

The authors wish to thank Prof. J.B. Gibson and Prof.
J.W.L. Kleevens for their encouragement and advice. We
are also grateful to Miss C. Yue for her excellent
secretarial assistance with records.

References

BELCHER, J.R. (1975). Adenocarcinoma and smoking.

Chest, 67, 622.

CHAN, W.C., COLBOURNE, M.J., FUNG, S.C. & HO, H.C.

(1979). Bronchial cancer in Hong Kong 1976-1977. Br.
J. Cancer, 39, 182.

CHAN, W.C. & MACLENNAN, R. (1977). Lung cancer in

Hong Kong Chinese: Mortality and histological types,
1960-1972. Br. J. Cancer, 35, 226.

CHINA (1979). Atlas of Cancer Mortality in the People's

Republic of China. China Map Press: Shanghai, China.

COX, J.D. & YESNER, R.A. (1979). Adenocarcinoma of the

lung: Recent results from the Veterans Administration
Lung Group. Am. Rev. Respir. Dis., 120, 1025.

DOLL, R., HILL, A.B. & KREYBERG, L. (1957). The

significance of cell type in relation to the aetiology of
lung cancer. Br. J. Cancer, 11, 43.

HIRAYAMA, T. (1981). Non-smoking wives of heavy

smokers have a higher risk of lung cancer: A study
from Japan. Br. Med. J., 282, 183.

HIRAYAMA, T. (1983). Passive smoking and lung cancer:

Consistency of association. Lancet, ii, 1425.

HONG KONG (1960-1981). Annual Report Medical &

Health Department. Hong Kong Government Printer.

KENNEDY, A. (1973). Relationship between cigarette

smoking and histological type of lung cancer in
women. Thorax, 28, 204.

KREYBERG, L. (1962). Histological lung cancer types: A

morphological and biological correlation. Acta. Pathol.
Microbiol. Scand., 157, (Suppl.), 1.

LAM, W.K., SO, S.Y. & YU, D.Y.C. (1983). Clinical features

of bronchogenic carcinoma in Hong Kong. Cancer, 52,
369.

LAW, C.H., DAY, N.E. & SHANMUGARATNAM, K. (1976).

Incidence rates of specific histological types of lung
cancer in Singapore Chinese dialect groups and their
aetiological significance. Int. J. Cancer, 17, 304.

LEE, S.H. & T'SO, T.O.T. (1963). Histological typing of lung

cancers in Hong Kong. Br. J. Cancer, 17, 37.

LEUNG, J.S.M. (1977). Cigarette smoking, the kerosene

stove and lung cancer in Hong Kong. Br. J. Dis.
Chest, 71, 273.

388    I.T.M. KUNG et al.

MACLENNAN, R., DA COSTA, J., DAY, N.E., LAE, C.H.,

NG, Y.K. & SHANMUGARATNAM, K. (1977). Risk
factors for lung cancer in Singapore Chinese, a
population with high female incidence rates. Int. J.
Cancer, 20, 854.

SCHOENTAL, R. & GIBBARD, S. (1967). Carcinogens in

Chinese incense smoke. Nature, 216, 612.

SEGI, M., AOKI, K. & KURIHARA, M. (1981). World

cancer mortality. In: Cancer Mortality and Morbidity
Statistics, (Eds. Segi et al.) Tokyo: Japan Scientific
Society Press, p. 121.

TRICHOPOULOS, D., KALANDIDI, A., SPARROS, L. &

MACMAHON, B. (1981). Lung cancer and passive
smoking. Int. J. Cancer, 177, 1.

VALAITIS, J., WARREN, S. & GAMBLE, D. (1981).

Increasing incidence of adenocarcinoma of the lung.
Cancer, 47, 1042.

VINCENT, R.G., PICKREN, J.W., LANE, W.W. & 5 others.

(1977). The changing histopathology of lung cancer.
Cancer, 39, 1647.

WATERHOUSE, J., MUIR, C., CORREA, P. & POWELL, J.

(Eds.). (1976). -Cancer Incidence in Five Continents,
V.III. Lyon: IARC Sci. Publ. No. 15.

WORLD HEALTH ORGANIZATION. (1967). Histological

Typing of Lung Tumours. (Eds. Kreyberg et al.) WHO,
Geneva.

WORLD HEALTH ORGANIZATION. (1981). Histological

Typing of Lung Tumours. WHO, Geneva.

				


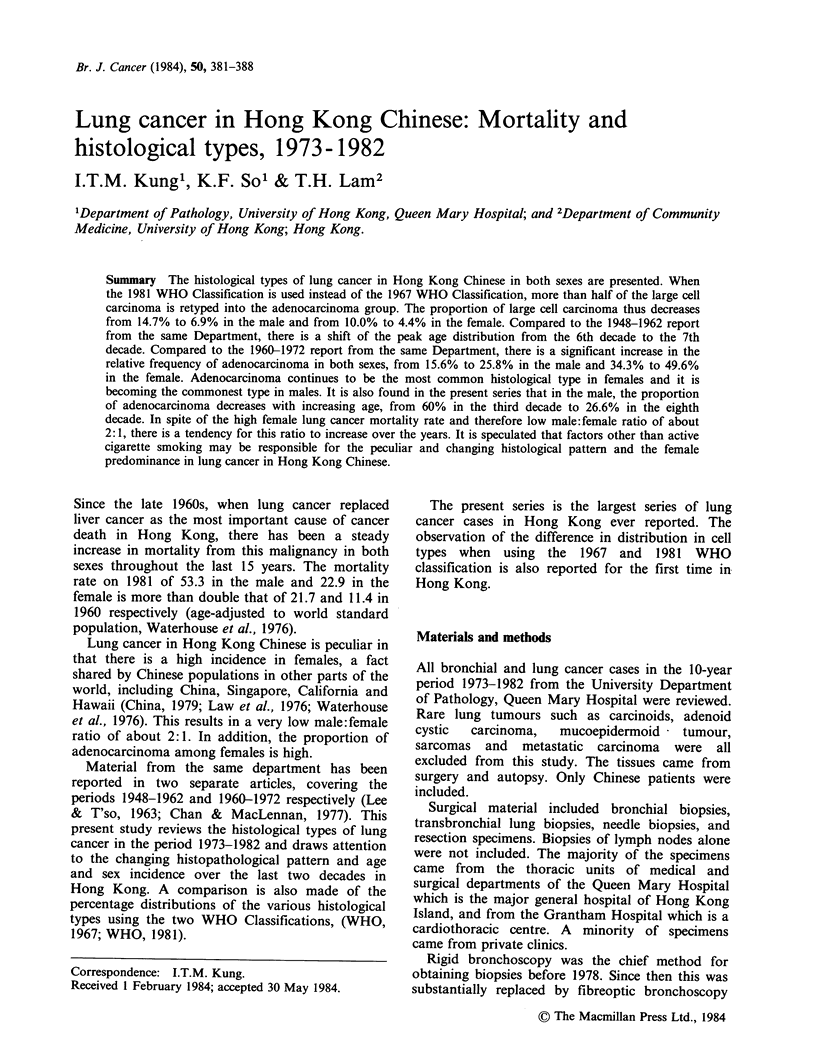

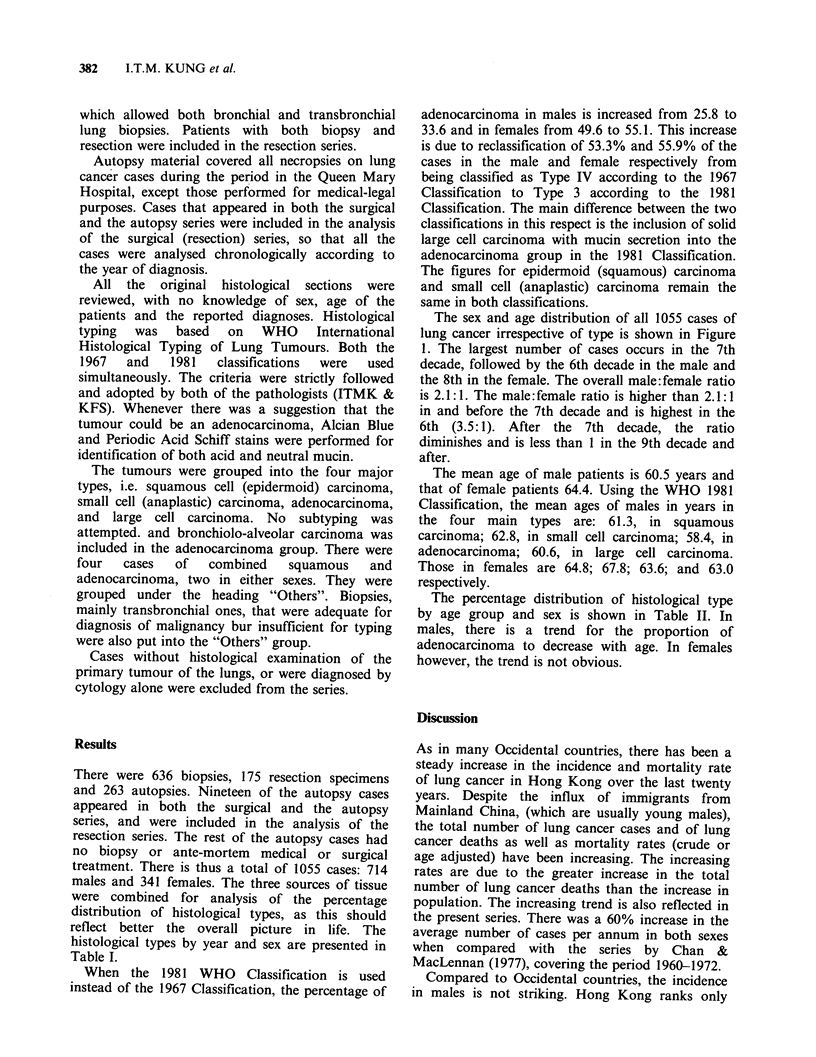

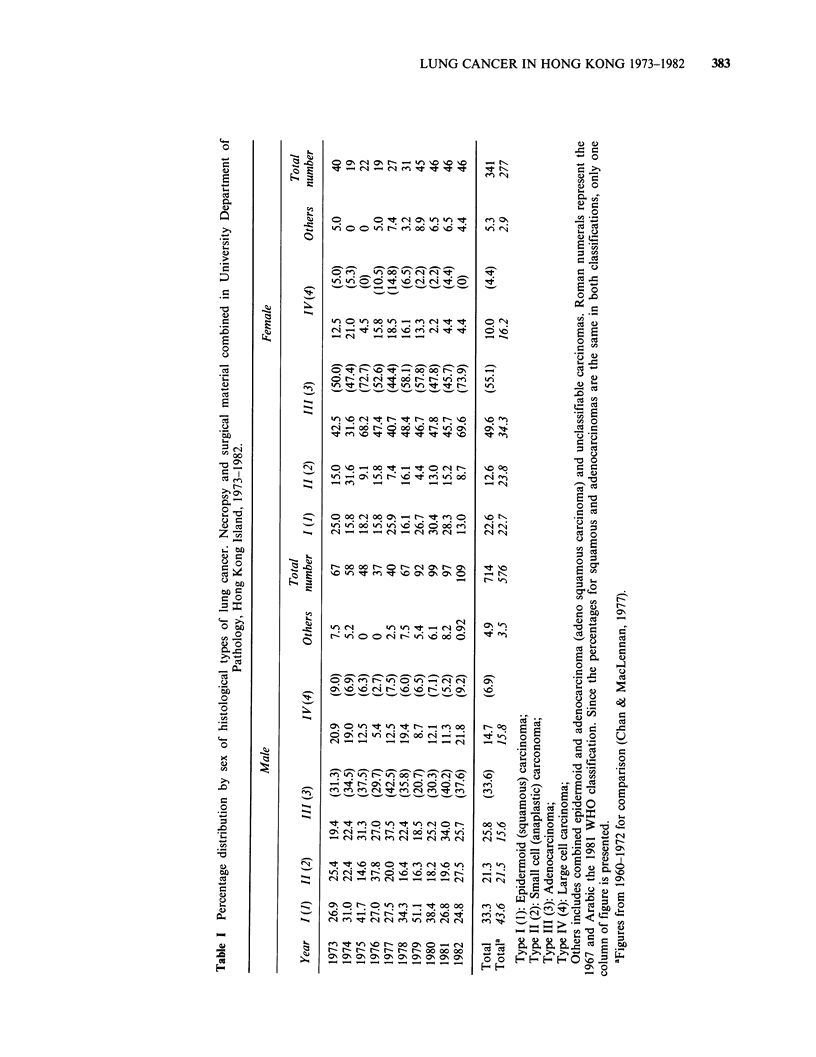

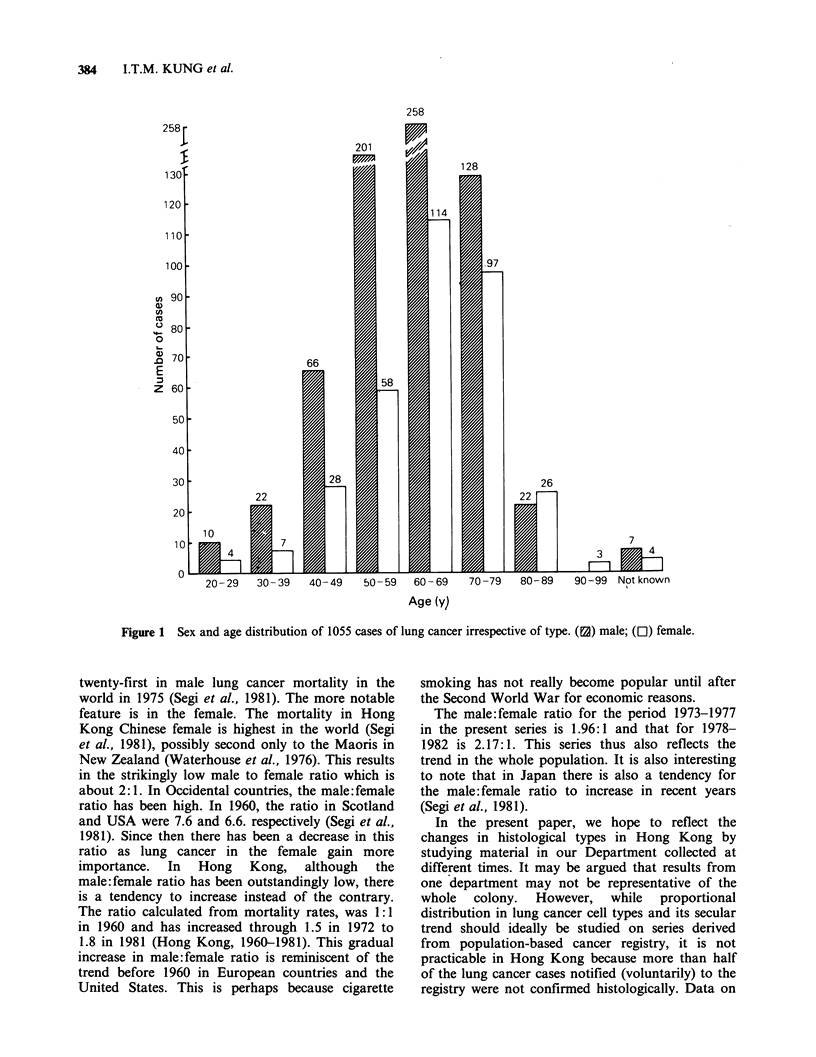

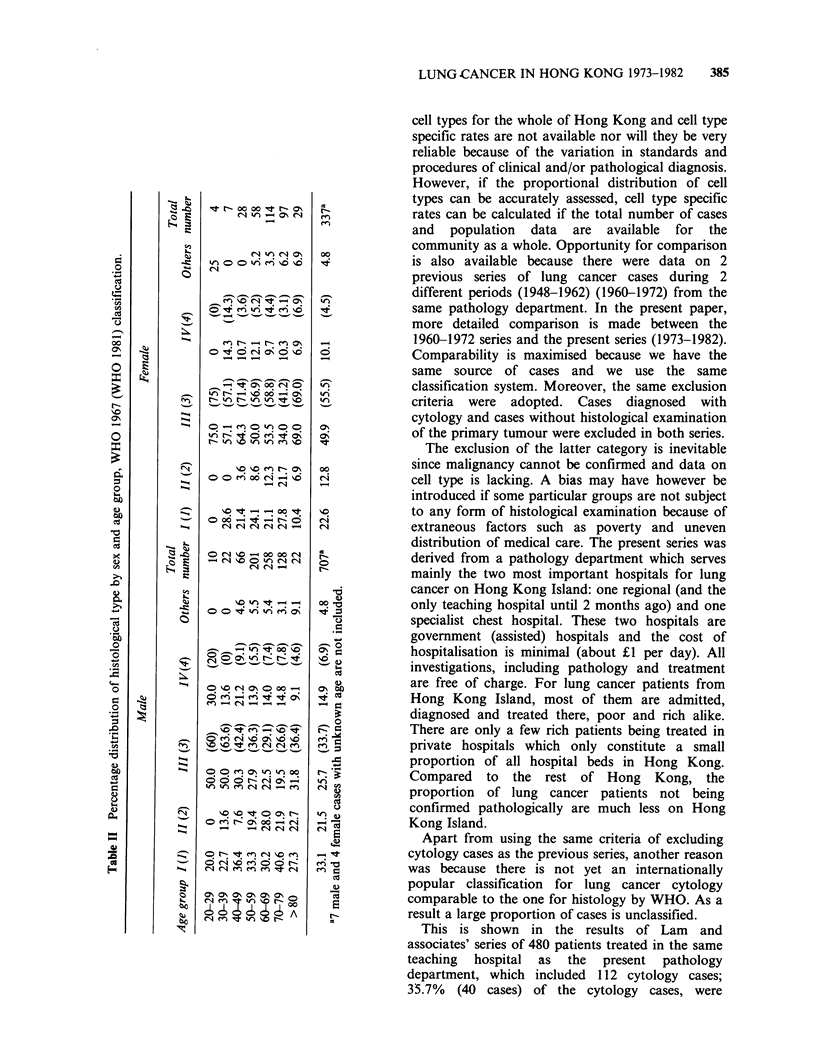

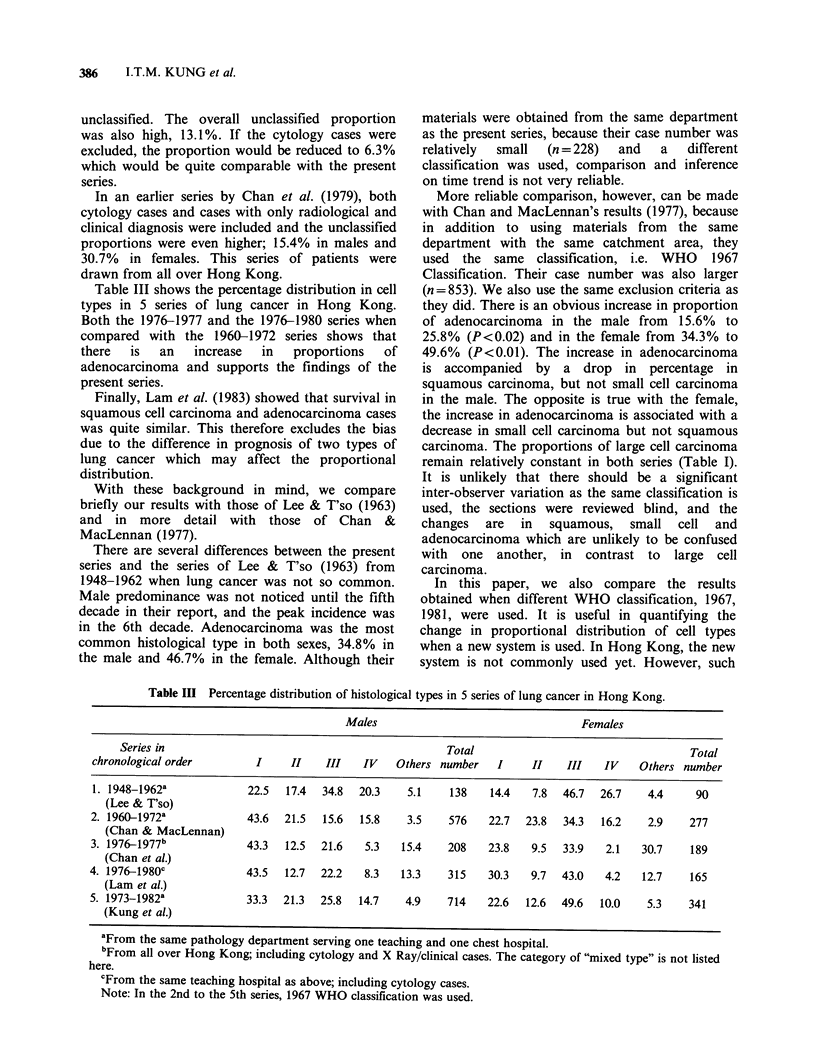

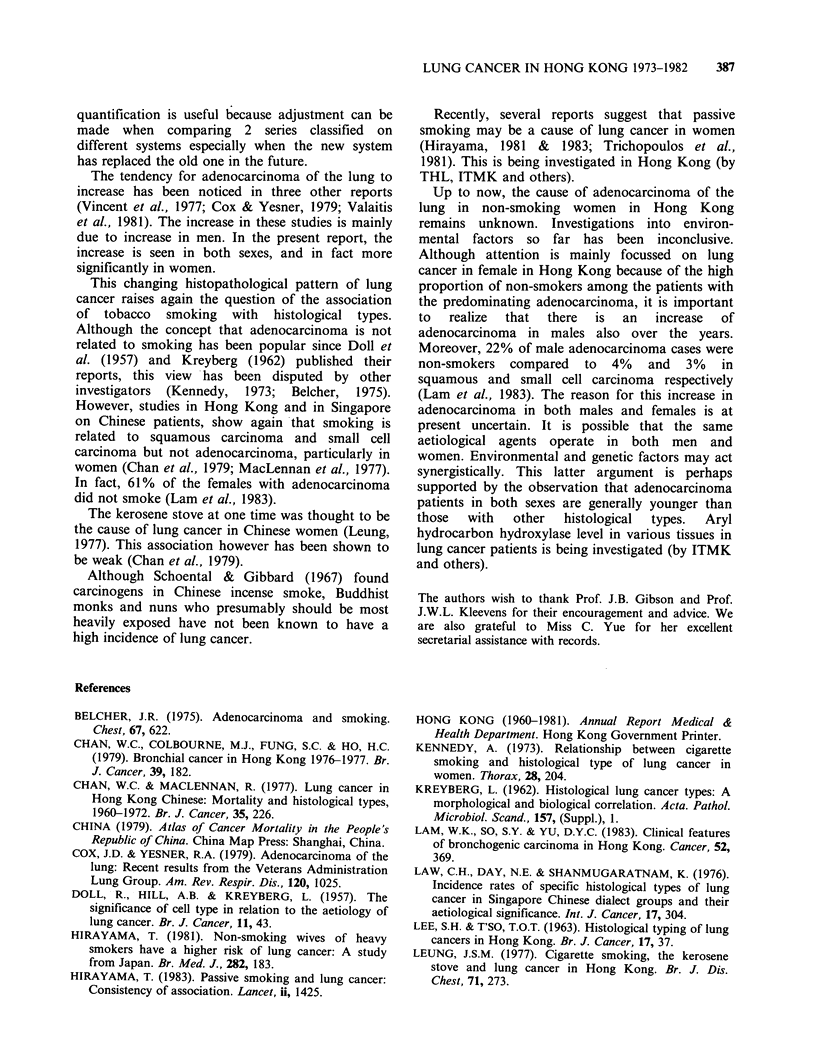

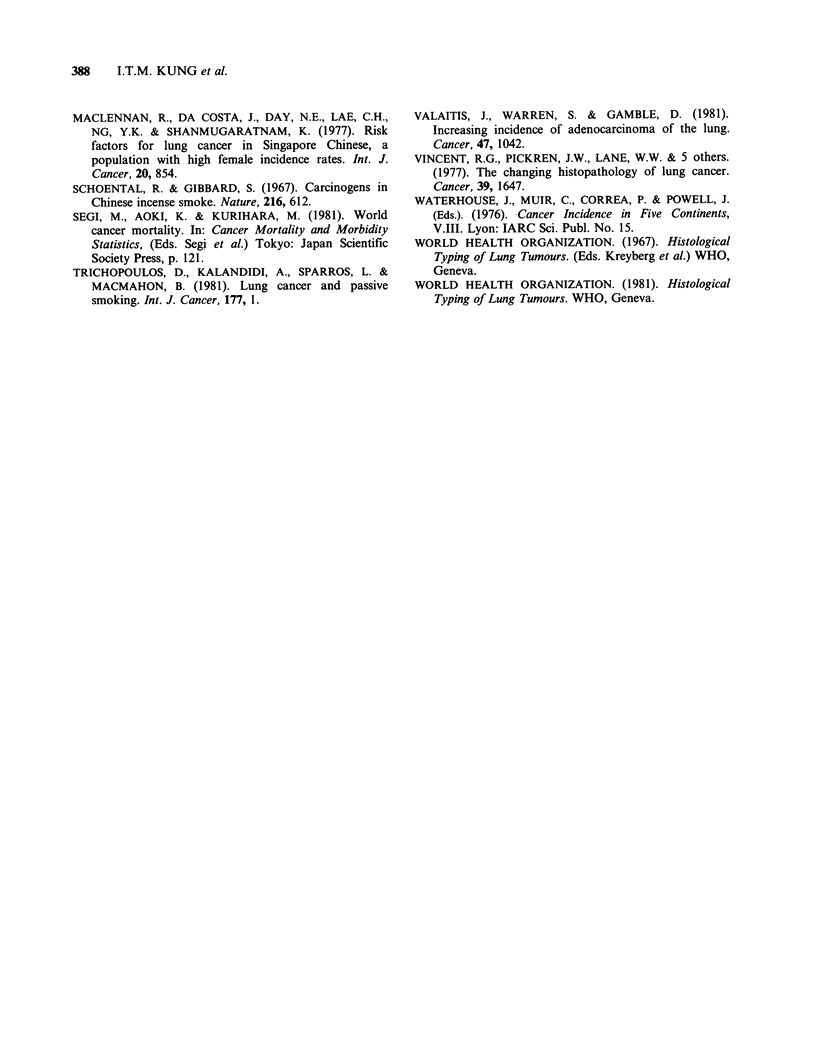

